# Disrupted Value-Directed Strategic Processing in Individuals with Mild Cognitive Impairment: Behavioral and Neural Correlates

**DOI:** 10.3390/geriatrics7030056

**Published:** 2022-05-11

**Authors:** Lydia T. Nguyen, Elizabeth A. Lydon, Shraddha A. Shende, Daniel A. Llano, Raksha A. Mudar

**Affiliations:** 1 Neuroscience Program, University of Illinois Urbana-Champaign, 405 North Mathews Ave, Urbana, IL 61801, USA; lydia.nguyen9@gmail.com (L.T.N.); d-llano@illinois.edu (D.A.L.); 2 Department of Speech and Hearing Science, University of Illinois Urbana-Champaign, 901 South 6th Street, Champaign, IL 61820, USA; elydon2@illinois.edu (E.A.L.); sshende2@illinois.edu (S.A.S.); 3 Department of Molecular and Integrative Physiology, University of Illinois Urbana-Champaign, 407 South Goodwin Ave, Urbana, IL 61801, USA; 4 Beckman Institute for Advanced Science and Technology, 405 North Mathews Ave, Urbana, IL 61801, USA; 5 Carle Neuroscience Institute, 610 N Lincoln Ave, Urbana, IL 61801, USA

**Keywords:** alpha, EEG, mild cognitive impairment, strategic processing, theta

## Abstract

Value-directed strategic processing involves attending to higher-value information while inhibiting lower-value information. This preferential processing is relatively preserved in cognitively normal older adults but is impaired in individuals with dementia. No studies have investigated whether value-directed strategic processing is disrupted in earlier stages of cognitive decline, namely, mild cognitive impairment (MCI). The current study examined behavioral and EEG differences in value-directed strategic processing between 18 individuals with MCI and 18 cognitively normal older controls using a value-directed list learning task. Behaviorally, individuals with MCI recalled fewer total and high-value words compared to controls, but no group differences were observed in low-value word recall. Neurally, individuals with MCI had reduced theta synchronization relative to controls between 100 and 200 ms post-stimulus. Greater alpha desynchronization was observed for high- versus low-value words between 300 and 400 ms in controls but not in the MCI group. The groups showed some processing similarities, with greater theta synchronization for low-value words between 700 and 800 ms and greater alpha desynchronization for high-value words between 500 and 1100 ms. Overall, value-directed strategic processing was compromised in individuals with MCI on both behavioral and neural measures relative to controls. These findings add to the growing body of literature on differences between typical cognitive aging and MCI.

## 1. Introduction

We are surrounded by vast amounts of information at any given moment, but this information differs in its value, importance, or relevance. The ability to attend to information of higher value while inhibiting information of lower value is referred to as value-directed strategic processing. This preferential processing ability minimizes cognitive burden and enables us to carry out everyday activities efficiently [1]. Evidence suggests that value-directed strategic processing is relatively well-preserved with normal cognitive aging [2,3,4,5]; however, the impact of cognitive impairment on strategic processing has been examined less frequently [6,7].

Two studies have examined value-directed strategic processing and recall in older adults with cognitive impairment, both of which focused on individuals with dementia, specifically Alzheimer’s dementia (AD) and behavioral variant frontotemporal dementia (bvFTD) [6,7]. Castel et al. [6] used a value-directed remembering (VDR) task with individuals with very mild and mild AD and cognitively normal younger and older controls. The VDR task utilized multiple word lists where the words were paired with different point values ranging between 1 and 12 points (e.g., Desk 12; Berry 1) and presented sequentially. At the end of each list, participants were asked to recall as many words from the list as they could with the goal of scoring maximal points. Overall, individuals with AD recalled fewer words compared to both cognitively normal younger and older adults across all word lists. While individuals with AD recalled more high- than low-value words, similar to cognitively normal younger and older adults, the magnitude of the difference between high- and low-value words recalled was significantly smaller in the AD group compared to the control groups. These findings suggest that although individuals with AD exhibit some level of value-directed strategic processing, their ability is compromised relative to cognitively normal older adults.

In the second study involving individuals with dementia, Wong et al. [7] contrasted the performance of individuals with AD and bvFTD with cognitively normal older adults using a simplified version of a VDR task. In this simplified VDR task, the same word list was repeated three times, similar to a typical episodic list learning task (e.g., the California Verbal Learning Test). The words were either low- (1 point), medium- (5 points), or high-value (10 points). Both individuals with AD and bvFTD recalled fewer words compared to cognitively normal older adults but performed comparably to one another. With regard to value-based recall, individuals with AD and bvFTD differed from cognitively normal older adults and from one another. The cognitively normal older adults demonstrated typical value-directed strategic processing (i.e., high- > medium- > low-value words recalled) across all lists. Individuals with AD showed some evidence for value-directed strategic processing with the third list (i.e., high- > medium- and low-value words recalled), but the individuals with bvFTD did not demonstrate this ability with any of the three lists (i.e., similar recall for high-, medium-, and low-value words). These findings suggest that strategic processing is differentially impaired across various types of dementia. To date, no studies have examined whether strategic processing based on value begins to deteriorate in earlier stages of cognitive decline, namely, mild cognitive impairment (MCI).

MCI is characterized by a decline in one or more cognitive domains that is greater than what is expected for a person’s age and education level but does not lead to significant functional decline, which would warrant a diagnosis of dementia [8,9,10]. It is well established that older adults with MCI are at higher risk of developing dementia compared to their cognitively normal peers [9,11,12,13]. While studies have largely focused on characterizing episodic memory deficits in individuals with MCI [14,15,16,17,18,19,20,21,22,23,24], growing evidence suggests impairments in other cognitive domains, including those relevant to value-directed strategic processing, namely, attention and inhibition.

Studies have shown impairment in a variety of attention tasks, including sustained, divided, and selective attention tasks, in individuals with MCI [25,26,27,28,29,30,31,32]. Deficits in inhibition have also been observed across various tasks, including Stroop, flanker, Hayling, Wisconsin Card Sorting Test, and stop-signal tasks [25,33,34,35,36,37,38]. Given these deficits in attention and inhibition, both of which are important for supporting value-directed strategic processing [1], one would anticipate challenges with value-directed strategic processing in individuals with MCI relative to cognitively normal older adults.

When examining cognitive alterations in MCI, such as value-directed strategic processing, it is beneficial to use neurophysiological measures as they capture early neural changes that may precede overt behavioral changes [39,40]. Event-related measures derived from electroencephalography (EEG) are well-suited for this purpose as they elucidate the neurophysiological underpinnings and temporal unfolding of cognitive processes with millisecond-level precision. Event-related spectral perturbations (ERSPs) are particularly useful to capture both spectral and temporal information about oscillatory brain activity in the EEG signal. ERSPs are typically discussed in terms of five different frequency bands, specifically, delta (1–4 Hz), theta (4–8 Hz), alpha (8–12 Hz), beta (12–30 Hz), and gamma bands (> 30 Hz). Power can then be estimated within each of these bands relative to a pre-stimulus baseline period and can either be more positive (event-related synchronization) or more negative (event-related desynchronization) relative to baseline [41]. Our previous work examined ERSPs during a value-directed word list learning task, with findings indicating that changes in theta and alpha band spectral power are linked to value-directed strategic processing [5,42]. In particular, we found greater synchronization in frontal theta for low- compared to high-value words and greater desynchronization in parietal alpha for high- compared to low-value words for both cognitively normal younger and older adults [5,42].

Task-related theta and alpha oscillations in individuals with MCI, while limited, have demonstrated differences in theta and alpha band power between individuals with MCI and cognitively normal older adults across a variety of tasks (e.g., *n*-back, Go/NoGo, Sternberg, simple attention/detection, attention orienting tasks) [43,44,45,46,47,48,49]. However, no studies have examined power differences in theta and alpha bands between individuals with MCI and cognitively normal older adults in the context of value-directed strategic processing. Thus, this study examined whether older adults with MCI have behavioral deficits and ERSP alterations during value-directed strategic processing when compared with cognitively normal older controls (CNCs) on the word list learning task used in our previous work [5,42]. For the behavioral data, we hypothesized that individuals with MCI would demonstrate impaired behavioral performance (i.e., recall fewer total words and fewer high-value words relative to CNCs) and neural alterations (i.e., differences in theta and alpha band power compared to CNCs).

## 2. Materials and Methods

### 2.1. Participants

Eighteen CNC participants and 18 older adults diagnosed with MCI participated in the study (see Table 1 for demographics). All participants were native English speakers, right-handed, and had a minimum high school level education. Individuals of both sexes were included, and no exclusions were made based on racial or ethnic factors. Participants had no history of stroke, dementia, Parkinson’s disease, traumatic brain injury, major psychiatric illness, epilepsy, alcohol or substance abuse, uncontrolled diabetes, autoimmune disease, learning disabilities, attention deficit hyperactivity disorder, or uncorrected vision or hearing loss.

The MCI participants had a clinical diagnosis of MCI from a dementia-specialist neurologist at the Carle Neuroscience Institute in Urbana, IL. All MCI participants met the clinical MCI guidelines of the 2011 US National Institute on Aging and Alzheimer’s Association workgroup [8], including: (a) cognitive concerns reported by the patient and/or corroborated by a reliable informant; (b) objectively verified impairments in one or more cognitive domains; (c) relative independence in activities of daily living; and (d) did not meet the criteria for dementia. The pattern of cognitive impairments in the MCI participants showed predominant impairment in memory, with declines in other cognitive domains, falling into the multi-domain MCI definition [50,51,52]. All participants in the MCI group completed the Clinical Dementia Rating [53] and received scores of 0.5. CNC participants had no subjective memory or cognitive complaints and performed normally on the cognitive assessments.

All participants completed a global cognitive screening followed by a more detailed cognitive assessment (Table 1). Global cognitive screening was completed using either the Mini-Mental State Evaluation (MMSE) [54] or the Montreal Cognitive Assessment (MoCA) [55]. All 18 CNC participants completed the MoCA and scored within the normal range (26 or above). Fourteen MCI participants completed the MoCA and four MCI participants completed the MMSE. The MMSE scores of the four MCI participants were converted to MoCA scores following the guidelines provided by Bergeron et al. [56] to create group averages. None of the participants reported elevated depressive symptoms (scored 5 or less on the Geriatric Depression Scale—Short form [57] or scored 10 or less on the Beck Depression Inventory [58]). Written informed consent was obtained from all participants in accordance with the protocols of both the University of Illinois Urbana-Champaign and the Carle Institutional Review Boards (protocol code 13191) before completing the study.

### 2.2. Strategic Processing Task and Procedures

All participants completed a value-directed word list learning task where the stimuli were 200 monosyllabic four-letter nouns from the MRC Psycholinguistic Database [59] and SUBTLEX_US_ database [60]. The 200 words were separated into 5 lists with 40 unique words per list. Each list contained a unique set of words in order to evaluate strategic processing, which differs from typical episodic list learning tasks (e.g., the California Verbal Learning Test) which repeat the same words across lists to examine episodic learning. Word stimuli were controlled for frequency, imageability, concreteness, and familiarity, and were comparable across lists. Additional task details are described in Nguyen et al. [5].

In each list, half of the words were assigned as high-value (*n* = 20) and were worth 10 points, and half were assigned as low-value (*n* = 20) and were worth 1 point. Letter case was used to differentiate high- and low-value words, such that half of the words were shown in all uppercase letters (e.g., LAMB) and half were shown in all lowercase letters (e.g., lamb). Font size was modified to ensure that all words appeared the same size on the screen regardless of letter case. Four versions of the task were created to counterbalance word value and letter case; two versions had high-value words in uppercase letters and low-value words in lowercase letters, and two versions had low-value words in uppercase letters and high-value words in lowercase letters. Participants were randomly assigned to one of the four task versions.

Participants were shown the following instructions on the screen: “You will see words appear on the screen one at a time. Some words are in uppercase and some words are in lowercase. The uppercase words *[lowercase words]* are worth 10 points each (high-value words). The lowercase words *[uppercase words]* are worth 1 point each (low-value words). At the end of the list, you will see the word “REMEMBER” on the screen. Your task is to remember as many of the words from the list as possible with the goal of scoring the maximum number of points. This is similar to a game in which words are worth different amounts of money”. Participants’ comprehension of the point values for the uppercase and lowercase words (dependent on the task version) was verified before starting the task. Participants were not provided with any instructions about how to be strategic, such as only memorizing the high-value words.

**Figure 1:**
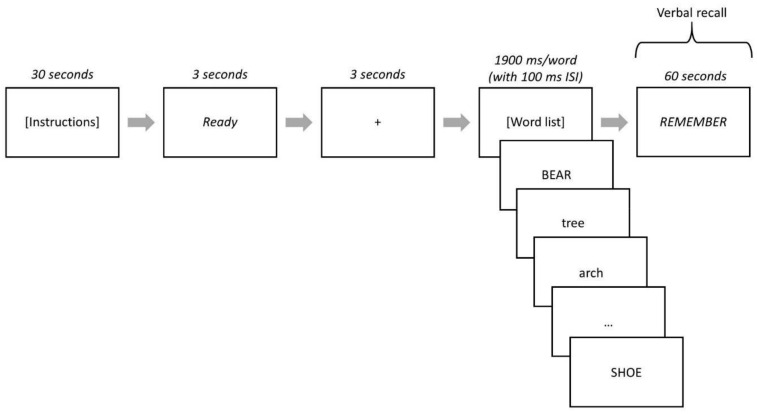
Strategic Processing Task Schematic. High- and low-value words were represented by lowercase or uppercase words depending on the task version. When the word “REMEMBER” was presented, participants verbally recalled words from the list and their responses were recorded on paper and scored. This process was repeated for all five lists.

After the instructions and confirmation of participant comprehension, the word “Ready” was presented at the center of the screen for 3000 ms followed by a fixation point (+) for 3000 ms. The word stimuli from a list were then individually presented at the center of the screen for 1900 ms each with an inter-stimulus interval of 100 ms (blank screen) between each word. After all 40 words in a list were presented, the word “REMEMBER” was presented at the center of the screen. Participants then had 60 s to verbally recall words from the list (see Figure 1 for task schematic) and their responses were manually recorded on a score sheet. Immediately following their verbal recall, participants received their score for the list before the next list was presented. After completion of all five lists, participants completed a brief interview about strategy use, if any, during the task (e.g., grouping words by category or remembering rhyming words).

### 2.3. EEG Data Collection and Preprocessing

A 64-electrode lycra cap (Neuroscan Quikcap) was used to record continuous EEG during the presentation of all five lists (a single testing session). A Neuroscan SynAmpsRT amplifier and Scan v4.5 software (sampling rate: 1 kHz, bandpass filter: DC-200 Hz) was used. Impedances typically were maintained below 10 kΩ. The reference electrode was located at the midline between Cz and CPz. Sites above and below the left eye were used to record vertical electrooculograms. Neuroscan Edit was used for offline preprocessing of the raw EEG data. EEG data from the five word lists (*n* = 200 stimuli) were appended for analysis in order to have a sufficient number of trials for each word value type (100 high-value trials; 100 low-value trials). Electrodes were excluded from further analysis if they were determined to be poorly functioning based on either high impedance values (above 20 kΩ) or visual inspection of raw EEG signals (on average, 1.8 electrodes per CNC were excluded and 1.2 electrodes per individual with MCI were excluded). Spatial filtering was used to correct eye blinks. EEG data were epoched from 500 ms pre-stimulus onset to 1500 ms after stimulus onset (i.e., −500 to 1500 ms). Epochs were rejected if their peak signal amplitude was ±75 μV. The rejection rates for high-value epochs were 18% and 21% for CNC and MCI groups, respectively, with no significant group difference; *F*(1,35) = 0.72, *p* = 0.403. The rejections rates for low-value epochs were 18% and 22% for CNC and MCI groups, respectively, with no significant group difference; *F*(1,35) = 1.52, *p* = 0.226. The EEG data were re-referenced to the average potential across the whole scalp.

### 2.4. ERSP Analysis

ERSPs were analyzed using the EEGLAB toolbox (Version 14.1.1b) [61] running on Matlab 2019a (MathWorks, Natick, MA, USA) from 0 to 1300 ms (post-stimulus onset) with a non-overlapping baseline from −400 to −100 ms (pre-stimulus onset). A short-time Fourier transform with Hanning window tapering was used for time-frequency decomposition through the EEGLAB function *newtimef.m*. A 256 ms sliding window and a pad ratio of 4 were used to give a frequency resolution of approximately 1 Hz. Gain model baseline correction was utilized, where each time-frequency point was divided by the average pre-stimulus baseline power at the same frequency [61,62].

### 2.5. ERSP Power Estimation

Mean spectral power was computed for the theta band (4–8 Hz) at two separate frontal electrode sites (Fz; FCz) and for the alpha band (8–12 Hz) at two separate parietal electrode sites (CPz; Pz). Mean power was computed for each group (CNC/MCI), value (high-/low-value), and frequency band (theta, alpha) in 13 time windows —100 ms sequential time windows from 0 ms to 1300 ms post-stimulus onset. Changes in power are described as synchronization or desynchronization, based on whether there was a power increase or decrease, respectively, relative to baseline. Traditional alpha band was used, as examination of individual alpha frequency (IAF) did not reveal any significant between-group differences for high-value words (*F*(1,35) = 1.47, *p* = 0.233) or low-value words (*F*(1,35) = 0.00, *p* = 1.000). The four electrode sites were chosen based on studies showing greater prominence of theta band at frontal sites and alpha at parietal/posterior sites [63,64,65,66] and on our previous studies with younger and older adults that used the same strategic processing task [5,42]. Individual midline electrodes were used to sample the data. Similar to our study, others have examined theta and alpha bands in individuals with MCI at individual electrodes, particularly midline electrodes, including the sites selected in the current study [45,67,68,69,70].

### 2.6. Statistical Analysis

IBM SPSS Statistics 28 was used for analysis. The behavioral data were first analyzed to determine whether there were significant differences between task version and word value (i.e., words in uppercase being assigned a high value vs. words in lowercase being assigned a high value). No significant differences were observed across versions (*p* > 0.05 for all five lists; see Table S1 in the Supplementary Materials for exact *p*-values); therefore, we combined data from both version types. A general linear model (GLM) was used to analyze task-related behavioral data, namely, the average number of high- and low-value words recalled, with group (CNC/MCI) as a between-subject factor and value (high-/low-value) as a within-subject factor.

Separate GLMs for theta and alpha bands were used to analyze ERSP data, with group (CNC/MCI) as a between-subject factor and value (high-/low-value) as a within-subject factor for each of the 13 time windows (100 ms time windows between 0 and 1300 ms post-stimulus onset). The Bonferroni method was used to correct for multiple comparisons with a threshold of *p* < 0.05. The reported *p*-values are derived from *F*- and *t*-statistics, unless specified otherwise.

## 3. Results

### 3.1. Task-Related Behavioral Data

**Figure 2:**
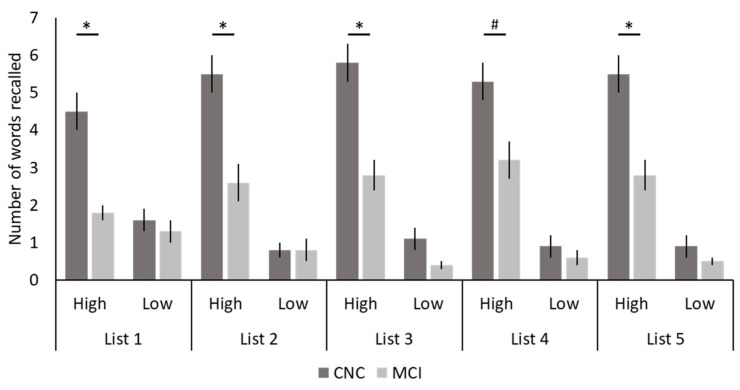
Task-Related Behavioral Data. The number of high- and low-value words recalled across the five lists for both cognitively normal older controls (CNCs) and individuals with mild cognitive impairment (MCI) are shown. Bars represent standard errors. * *p* < 0.05; # *p* = 0.070 (trending).

Task-related behavioral data showed significant main effects of group, with more total words recalled by CNCs than individuals with MCI for all five lists (*p* < 0.001), as well as significant main effects of value with more high- than low-value words recalled for all five lists (*p* < 0.001; see Table 2 for exact *p*-values). These main effects were qualified by significant interaction effects between group and value for Lists 1, 2, 3, and 5 (*p* < 0.01), and a trend for List 4 (*p* = 0.070; Table 2; Figure 2). Post hoc analyses revealed that for all five lists there were between-group differences for the high-value words, with more high-value words recalled by CNCs than individuals with MCI (List 1: *p* < 0.001; List 2: *p* < 0.001; List 3: *p* < 0.001; List 4: *p* = 0.005; List 5: *p* < 0.001), and no between-group differences for low-value words (List 1: *p* = 0.449; List 2: *p* = 1.000; List 3: *p* = 0.071; List 4: *p* = 0.402; List 5: *p* = 0.193).

### 3.2. Theta Band (4–8 Hz) Mean Power

For Fz, there were no significant main effects of group (*p* > 0.05; see Table 3 for exact *p*-values), no significant main effects of value (*p* > 0.05; see Table 4 for exact *p*-values), and no significant interaction effects between group and value (*p* > 0.05; see Table 5 for exact *p*-values) for any of the 13 time windows (100 ms time windows between 0 and 1300 ms post-stimulus onset).

**Figure 3:**
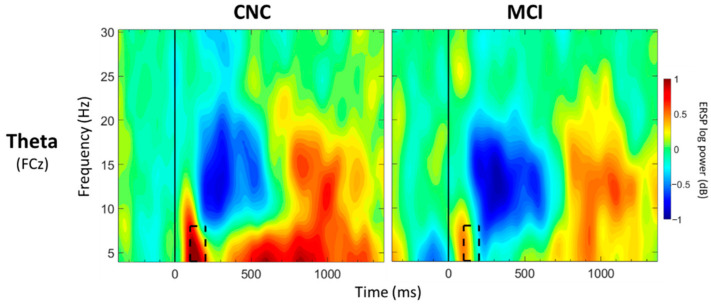
ERSP Comparisons for Main Effects of Group. Spectrograms illustrate differences between groups (CNC/MCI) for theta band (4–8 Hz) at FCz. The 0 ms time point (solid vertical line) represents stimulus onset. Dashed black rectangles indicate the time windows in which significant main effects of group were observed (also see Table 3). CNC: Cognitively normal older controls; MCI: mild cognitive impairment.

**Figure 4:**
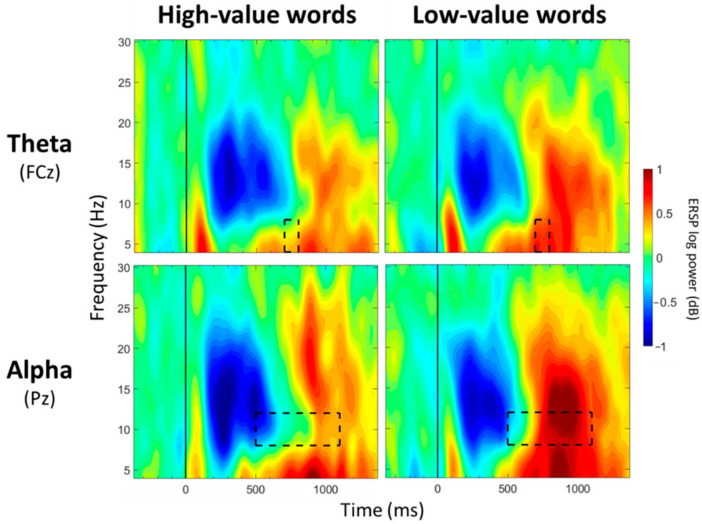
ERSP Comparisons for Main Effects of Value. Spectrograms illustrate differences between value (high-/low-value) for theta band (4–8 Hz) at FCz and alpha band (8–12 Hz) at Pz. The 0 ms time point (solid vertical line) represents stimulus onset. Dashed black rectangles indicate the time windows in which significant main effects of value were observed (also see Table 4).

**Figure 5:**
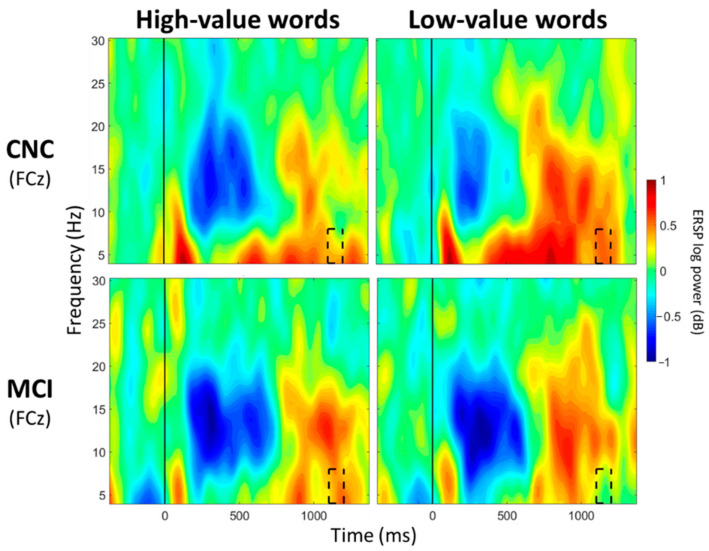
ERSP Comparisons for Theta Band for Interaction Effects Between Group and Value. Spectrograms illustrate differences between groups (CNC/MCI) and value (high-/low-value) for theta band (4–8 Hz) at FCz. The 0 ms time point (solid vertical line) represents stimulus onset. Dashed black rectangles indicate the time windows in which significant interaction effects between group and value were observed (also see Table 5). CNC: Cognitively normal older controls; MCI: mild cognitive impairment.

For FCz, a significant main effect of group was observed between 100 and 200 ms post-stimulus onset (*p* < 0.05; Table 3; Figure 3), with greater theta synchronization in the CNC group than in the MCI group. A significant main effect of value was observed between 700 and 800 ms post-stimulus onset (*p* < 0.05; Table 4; Figure 4), with greater theta synchronization for low- compared to high-value words. A significant interaction effect between group and value was observed between 1100 and 1200 ms post-stimulus onset (*p* < 0.05; Table 5; Figure 5); however, post hoc analyses did not reveal any between- or within-group differences (*p* > 0.05).

### 3.3. Alpha Band (8–12 Hz) Mean Power

**Figure 6:**
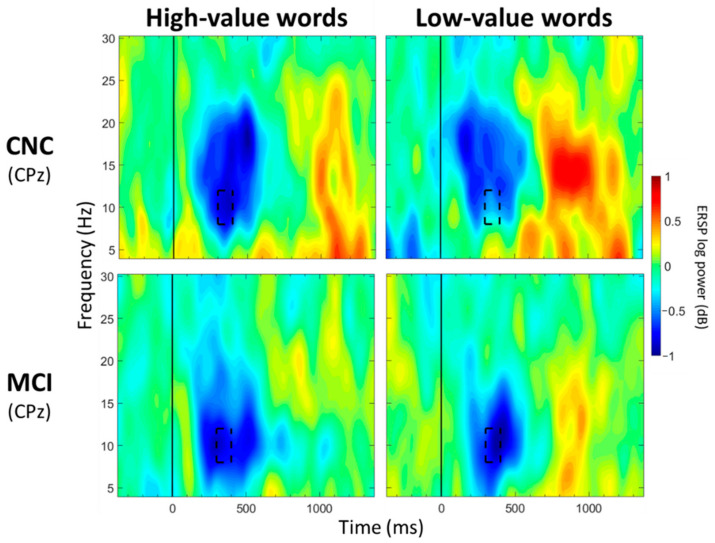
ERSP Comparisons for Alpha Band for Interaction Effects Between Group and Value. Spectrograms illustrate differences between groups (CNC/MCI) and value (high-/low-value) for alpha band (8–12 Hz) at CPz. The 0 ms time point (solid vertical line) represents stimulus onset. Dashed black rectangles indicate the time windows in which significant interaction effects between group and value were observed (also see Table 5). CNC: Cognitively normal older controls; MCI: mild cognitive impairment.

For CPz, no significant main effects of group were observed for any of the 13 time windows (*p* > 0.05; Table 3). Significant main effects of value were observed between 500 and 600 ms and between 700 and 1000 ms post-stimulus onset (*p* < 0.05; Table 4; Figure 4), with greater alpha desynchronization for high-compared to low-value words. A significant interaction effect between group and value was observed between 300 and 400 ms post-stimulus onset (*p* < 0.05; Table 5; Figure 6). Post hoc analyses did not reveal any between-group differences, but a within-group difference was observed for the CNC group (*p* = 0.021), with greater alpha desynchronization for high- than low-value words, but not for the MCI group (*p* = 0.648).

For Pz, no significant main effects of group were observed for any of the 13 time windows (*p* > 0.05; Table 3). Significant main effects of value were observed from 500 to 1100 ms post-stimulus onset (*p* < 0.05; Table 4; Figure 4), with greater alpha desynchronization for high- compared to low-value words. There were no significant interaction effects between group and value for any of the 13 time windows (*p* > 0.05; Table 5).

## 4. Discussion

The purpose of the current study was to examine value-directed strategic processing in older adults with MCI relative to CNCs. Differences between groups were found with respect to both behavioral and neural measures, while some similarities in value-directed strategic processing were also observed.

Behavioral data revealed lower recall of high-value and total number of words in the MCI group compared to the CNC group across all five lists. These findings were not surprising given the extensive literature from list learning tasks that show that deficits in episodic learning and memory are present in individuals with MCI [14,15,16,17,18,19,20,21,22,23,24]. Impaired recall of high-value words in the MCI group compared to the CNC group may stem from underlying deficits in episodic memory (encoding, storage, and retrieval). Studies supporting preserved strategic recall in cognitively normal older adults have posited that strategic encoding of higher-value items relative to lower-value items, as well as strategic retrieval of higher-value items before lower-value items, reduces the chance of forgetting more valuable information [71,72]. Individuals with MCI in the current study may have impairments in strategic encoding, strategic recall, or both. A recent review proposed a dual-mechanism framework of value-directed encoding defined by both intentional and automatic processes [71]. The intentional processes are characterized by deeper semantic processing and encoding strategies, whereas automatic processes are characterized by preferential processing of rewarding or salient information. During exit interviews following our task, only 61% of individuals with MCI reported using a strategy to perform the task compared to 83% of CNCs. This aligns with findings from list learning studies showing reduced strategy use in individuals with MCI compared to CNCs [22,73,74,75]. Thus, the relative lack of strategy use in the MCI group compared to the CNC group, in conjunction with possible declines in automatic processes, may also have hindered their ability to selectively process and encode high-value words.

Behaviorally, there were no significant group differences in the number of low-value words recalled, which could indicate that the individuals with MCI were still able to strategically inhibit, or forget, the low-value words. However, it may also be the case that the individuals with MCI forgot the low-value words. The use of a value-directed directed forgetting paradigm with a recognition test [72,76] in future studies could help elucidate whether individuals with MCI are indeed inhibiting the low-value information (i.e., recognize the to-be-forgotten items) or whether they have forgotten the items (i.e., would not recognize the to-be-forgotten items). It is also likely that our sample size was small for capturing group differences in low-value word recall given that, typically, fewer low-value words are recalled relative to high-value words. Overall, the behavioral data provide some indication of impaired strategic processing of high-value words, but these data are confounded by memory processes (i.e., encoding, storage, and retrieval). Thus, ERSPs, which allow for an examination of cognitive processing in real-time at the moment of stimulus presentation, are particularly useful for better understanding the behavioral findings.

ERSP differences between the CNC and MCI groups were observed in both theta and alpha bands. Reduced frontal theta synchronization was observed in the MCI group compared to the CNC group between 100 and 200 ms (FCz) post-stimulus onset. The current findings are consistent with a handful of studies that have observed reduced theta synchronization in MCI participants compared to CNC participants across various tasks (i.e., Go/NoGo, *n*-back, and Sternberg tasks) [44,45,46,48,49]. Frontal theta synchronization has been linked to inhibitory processes [63,77,78]. The MCI group may not have been able to engage early inhibitory processes at the same level as the CNC group [25,33,34,36,37,38]. The group-by-value interaction observed in the alpha band between 300 and 400 ms (CPz) post-stimulus onset lends support to the notion that the MCI group may process high- and low-value words more similarly than the CNC group. Although the CNC group demonstrated greater alpha desynchronization for high- compared to low-value words, the MCI group did not show these differences, suggesting a lack of neural differentiation in processing information of varying values (i.e., similar processing for both high- and low-value words). Whether this lack of neural differentiation in alpha band power is related to early changes in the neural substrates that support the intentional processes, i.e., semantic processing as proposed by Knowlton and Castel [71], needs further examination. Given the link between alpha band and semantic processing [79,80], combined functional magnetic resonance imaging (fMRI) and EEG would be valuable to examine this relationship. Additionally, recent fMRI evidence suggests that semantic processing regions, including left superior temporal gyrus and left lateral temporal cortex, are engaged in older adults during the processing of higher-value words [81]. A diffusion tensor imaging study has also shown that preferential recall of high-value words in older adults was dependent on the integrity of the inferior fronto-occipital fasciculus, a tract associated with semantic memory performance and retrieval of semantic information [82]. These regions and tracts have been shown to be altered in individuals with MCI compared to CNCs [83,84,85,86,87,88]. Examining the links between neurophysiological alterations in alpha band and the neural substrates that support value-directed strategic processing in MCI will advance our theoretical understanding of value-directed strategic processing.

Despite group differences between the MCI and CNC groups, some similarities across the groups were observed in the processing of high- versus low-value words. Both groups showed greater theta synchronization for low- compared to high-value words from 700 to 800 ms (FCz) post-stimulus onset and greater alpha desynchronization for high- compared to low-value words between 500 and 600 and between 700 and 1000 ms (CPz) and between 500 and 1100 ms (Pz) post-stimulus onset. These findings are similar to our previous studies involving cognitively normal younger and older adults engaged on the same task [5,42]. The consistency in the distinct neural patterns observed for high- and low-value words across ages and cognitive statuses suggests that theta and alpha bands are robust neural measures of value-directed strategic processing. It was not unexpected to find some similarities between the groups for processing high- and low-value information as there is evidence that individuals with MCI retain some ability to extract important information, although they are still impaired relative to CNCs [89,90,91,92]. Additionally, Castel et al. [6] found that individuals with very mild and mild AD recalled more high- than low-value words, suggesting some degree of strategic processing.

Certain limitations of the current study need to be addressed in future work. First, our sample size was small. Given the heterogeneity observed in the MCI population, it would be helpful to validate these findings in a larger study. Second, it may be useful to use data reduction techniques such as principal component analysis to identify time points and electrodes of interest through a more data-driven (as opposed to hypothesis-driven) approach. Third, the current task was a passive task and was not designed to examine how subsequent recall may relate to value-directed strategic processing. Specifically, the task design did not allow us to examine how ERSP data during stimulus processing differs between words that were recalled successfully versus unsuccessfully, as is typical in subsequent memory paradigms [93,94,95]. Such a comparison might provide more clarity as to whether lower recall of high-value words in the MCI group relative to the CNC group was due to impairments in episodic memory or value-directed strategic processing. In addition, tracking the order of word recall could aid our understanding of strategic retrieval in individuals with MCI (e.g., do they recall high-value items first?) [72]. Lastly, studies that have used list learning tasks have shown reductions in spontaneous strategy use in cognitively normal older adults [96,97,98] and in individuals with MCI [22,73,74,75]. Providing explicit instructions to use strategies (e.g., grouping similar information) has been shown to improve recall in both cognitively normal older adults [99,100] and individuals with MCI [22]. As such, explicitly defining value based on conceptual information, such as categories (e.g., animals are high-value words), may have interesting effects on value-directed strategic processing for both CNC and MCI individuals and should be examined in future studies.

In conclusion, the current study showed that value-directed strategic processing is compromised both behaviorally and neurally in individuals with MCI as compared to CNCs. The group differences in theta and alpha bands suggest that the MCI and CNC groups regulated strategic processing differently, which may have contributed to impaired recall of high-value words in the MCI group relative to the CNC group. The similarities across MCI and CNC groups for theta and alpha bands showed that there are distinct neural markers linked to the processing of high- and low-value words. These distinct neural markers are consistent with what we have found in our previous studies [5,42], demonstrating the utility of ERSPs as measures of value-directed strategic processing across the spectrum of cognitive aging. The findings of the current study may be clinically applicable to understand differences between typical cognitive aging and MCI.

## Figures and Tables

**Table 1:** Participant Demographics and Cognitive Testing Performance.

	CNC (*n* = 18)	MCI (*n* = 18)	*p*-Value
*Demographics*			
Age (years)	74.5 (4.7)	76.7 (4.2)	0.146
Education (years)	16.3 (3.0)	16.0 (3.4)	0.795
Sex	15F/3M	15F/3M	1.00
*Cognitive Testing*			
Montreal Cognitive Assessment	27.4 (1.4)	21.3 (3.9)	<0.001 **
LM—Immediate (Story A)	15.9 (3.2) ^a^	8.2 (3.1) ^a^	<0.001 **
LM—Delayed (Story A)	14.1 (4.2) ^a^	4.0 (3.1) ^a^	<0.001 **
Letter fluency (F, A, S)	49.2 (8.8)	38.0 (14.4)	0.008 **
Category fluency (Animals)	19.9 (4.0)	13.9 (4.8)	<0.001 **
Boston Naming Test (30 items)	27.8 (1.6)	26.6 (2.5) ^a^	0.116
Trail Making Test A (s)	27.7 (6.9)	34.0 (16.6)	0.145
Trail Making Test B (s)	80.2 (29.0)	130.1 (59.1)	0.003 **
Digit span—forward	6.5 (1.5) ^a^	7.0 (1.2) ^a^	0.347
Digit span—backward	5.1 (1.2) ^a^	5.2 (1.3) ^a^	0.880

Cells represent mean (standard deviation). ^a^ *n* = 14. The *p*-values were derived from one-way ANOVAs, except for sex which was derived from Pearson chi-square testing. ** *p* < 0.01. CNC: cognitively normal older controls; MCI: mild cognitive impairment; LM: Wechsler Memory Scale IV Logical Memory subtest.

**Table 2:** Statistical Results for Task-Related Behavioral Data.

		Main Effect: Group	Main Effect: Value	Interaction: Group x Value
List 1	*F*(1,34) *p*	34.62 <0.001 **	17.61 <0.001 **	8.08 0.008 **
List 2	*F*(1,34) *p*	23.12 <0.001 **	51.85 <0.001 **	10.42 0.003 **
List 3	*F*(1,34) *p*	26.22 <0.001 **	83.32 <0.001 **	8.70 0.006 **
List 4	*F*(1,34) *p*	14.27 <0.001 **	54.22 <0.001 **	3.50 0.070
List 5	*F*(1,34) *p*	30.26 <0.001 **	67.89 <0.001 **	7.79 0.009 **

Cells display statistics for main effects of group (CNC/MCI), main effects of value (high-/low-value words), and interaction effects between group and value for the five word lists. ** *p* < 0.01.

**Table 3:** Statistical Results for Main Effects of Group for Theta and Alpha Band Mean Power.

		Time (ms)												
		0–100	100–200	200–300	300–400	400–500	500–600	600–700	700–800	800–900	900–1000	1000–1100	1100–1200	1200–1300
**Theta (4–8 Hz)**														
Fz	*F*(1,34) *p*	0.08 0.784	3.59 0.066	1.35 0.253	1.27 0.268	0.69 0.412	0.92 0.343	1.42 0.242	0.61 0.441	0.68 0.414	0.85 0.362	1.26 0.270	0.92 0.345	0.08 0.773
FCz	*F*(1,34) *p* $\eta_{p}^{2}$	0.28 0.600	**5.55** **0.024** **0.14**	3.13 0.086	3.65 0.065	2.98 0.093	1.78 0.192	1.86 0.181	0.74 0.395	0.78 0.384	0.32 0.577	0.91 0.346	0.66 0.423	0.69 0.413
**Alpha (8–12 Hz)**														
CPz	*F*(1,34) *p*	0.01 0.938	0.06 0.810	0.09 0.766	0.35 0.556	0.43 0.515	2.08 0.158	0.82 0.371	1.33 0.257	0.26 0.613	0.43 0.516	3.47 0.071	2.14 0.153	1.60 0.214
Pz	*F*(1,34) *p*	0.49 0.489	0.06 0.802	0.04 0.847	0.35 0.559	0.53 0.473	2.92 0.096	3.19 0.083	1.93 0.174	0.60 0.443	1.53 0.225	1.91 0.176	2.13 0.154	1.04 0.315

Cells display statistics for main effects of group (CNC/MCI) for mean power in theta band (4–8 Hz) at Fz and FCz electrodes and in alpha band (8–12 Hz) at CPz and Pz electrodes across 13 time windows post-stimulus onset. Significant main effects of value (*p* < 0.05, Bonferroni-corrected) are indicated by emboldened values and their effect sizes ($\eta_{p}^{2}$) are reported.

**Table 4:** Statistical Results for Main Effects of Value for Theta and Alpha Band Mean Power.

		Time (ms)												
		0–100	100–200	200–300	300–400	400–500	500–600	600–700	700–800	800–900	900–1000	1000–1100	1100–1200	1200–1300
**Theta (4–8 Hz)**														
Fz	*F*(1,34) *p*	0.59 0.446	0.06 0.805	0.00 0.977	0.42 0.519	0.57 0.454	1.94 0.172	2.43 0.129	3.13 0.086	3.20 0.083	1.87 0.180	1.28 0.266	0.54 0.468	0.58 0.452
FCz	*F*(1,34) *p* $\eta_{p}^{2}$	0.60 0.444	0.24 0.625	0.03 0.874	0.29 0.595	2.77 0.105	0.74 0.396	1.84 0.183	**6.21** **0.018** **0.15**	1.65 0.207	0.90 0.351	0.01 0.937	0.01 0.942	0.45 0.506
**Alpha (8–12 Hz)**														
CPz	*F*(1,34) *p* $\eta_{p}^{2}$	0.00 0.977	0.09 0.767	1.36 0.252	3.78 0.060	0.15 0.699	**7.81** **0.008** **0.19**	3.73 0.062	**13.34** **0.001** **0.28**	**11.45** **0.002** **0.25**	**6.90** **0.013** **0.17**	1.73 0.197	0.08 0.772	0.00 0.982
Pz	*F*(1,34) *p* $\eta_{p}^{2}$	0.47 0.495	0.00 0.971	0.97 0.332	0.00 0.973	0.00 0.973	**6.23** **0.018** **0.16**	**11.61** **0.002** **0.26**	**27.89** **<0.001** **0.45**	**36.07** **<0.001** **0.52**	**19.44** **<0.001** **0.36**	**5.93** **0.020** **0.15**	0.75 0.394	0.26 0.617

Cells display statistics for main effects of value (high-/low-value words) for mean power in theta band at Fz and FCz electrodes and in alpha band at CPz and Pz electrodes across 13 time windows post-stimulus onset. Significant main effects of value (*p* < 0.05, Bonferroni-corrected) are indicated by emboldened values and their effect sizes ($\eta_{p}^{2}$) are reported.

**Table 5:** Statistical Results for Group-by-Value Interactions for Theta and Alpha Band Mean Power.

		Time (ms)												
		0–100	100–200	200–300	300–400	400–500	500–600	600–700	700–800	800–900	900–1000	1000–1100	1100–1200	1200–1300
**Theta (4–8 Hz)**														
Fz	*F*(1,34) *p*	0.07 0.793	0.49 0.490	0.02 0.875	1.33 0.258	0.54 0.467	0.35 0.560	0.17 0.681	1.88 0.179	0.15 0.699	0.00 0.994	0.40 0.532	1.26 0.269	0.49 0.489
FCz	*F*(1,34) *p* $\eta_{p}^{2}$	0.17 0.681	0.40 0.533	2.15 0.152	2.01 0.166	2.60 0.116	2.09 0.157	0.76 0.391	2.17 0.150	0.89 0.352	0.17 0.681	0.00 0.989	**4.76** **0.036** **0.12**	0.34 0.566
**Alpha (8–12 Hz)**														
CPz	*F*(1,34) *p* $\eta_{p}^{2}$	0.20 0.662	0.02 0.902	0.94 0.339	**5.85** **0.021** **0.15**	0.95 0.336	0.12 0.733	0.03 0.857	0.29 0.592	0.10 0.757	0.40 0.530	1.22 0.276	0.03 0.854	0.02 0.886
Pz	*F*(1,34) *p*	1.17 0.287	0.10 0.755	0.03 0.864	3.18 0.084	3.09 0.088	2.42 0.129	0.97 0.331	2.01 0.165	2.37 0.133	0.11 0.747	0.29 0.593	0.03 0.870	0.00 0.992

Cells display statistics for interaction effects between group (CNC/MCI) and value (high-/low-value words) for mean power in theta band at Fz and FCz electrodes and alpha band at CPz and Pz electrodes across 13 time windows post-stimulus onset. Significant interaction effects between group and value (*p* < 0.05, Bonferroni-corrected) are indicated by emboldened values and their effect sizes ($\eta_{p}^{2}$) are reported.

## Data Availability

The data presented in this study are available on request from the corresponding author. The data are not publicly available for privacy reasons.

## Supplementary Materials

The following supporting information can be downloaded at: https://www.mdpi.com/article/10.3390/geriatrics7030056/s1, Table S1: Statistical Results for the Effects of Version on Behavioral Data.
