# Supplementary material for: Disrupted Value-Directed Strategic Processing in Individuals with Mild Cognitive Impairment: Behavioral and Neural Correlates
Source: Geriatrics (Basel). 2022 May 11;7(3):56. doi: 10.3390/geriatrics7030056 (PMC9149834; doi:10.3390/geriatrics7030056)
Supplement: Supplementary file 1 [file geriatrics-07-00056-s001.zip › geriatrics-1690961-supplementary.pdf]

Supplementary Material

Disrupted Value-Directed Strategic Processing in Individuals with Mild Cognitive Impairment: Behavioral and Neural Correlates

Lydia T. Nguyen, Elizabeth A. Lydon, Shraddha A. Shende, Daniel A. Llano, & Raksha A. Mudar

Table S1. Statistical Results for the Effects of Version on Behavioral Data.

		**Interaction: Version x Value**
**List 1**	$F(1,34)$	1.55
	p	.222
**List 2**	$F(1,34)$	2.48
	p	.124
**List 3**	$F(1,34)$	0.06
	p	.803
**List 4**	$F(1,34)$	0.01
	p	.912
**List 5**	$F(1,34)$	0.53
	p	.474

Cells display statistics for interaction effects between version (words in uppercase being assigned to high-value/words in lowercase being assigned to high-value) and value (high-value/low-value) for each of the five lists. There were no significant differences observed across versions.
